# Chronic Viral Infections and Cancer, Openings for Therapies and Vaccines

**DOI:** 10.3390/cancers16040818

**Published:** 2024-02-18

**Authors:** Maria G. Isaguliants, Alexander V. Ivanov, Franco M. Buonaguro

**Affiliations:** 1 Department of Microbiology, Tumor and Cell Biology, Karolinska Institutet, 171 77 Stockholm, Sweden; 2 Engelhardt Institute of Molecular Biology, Russian Academy of Sciences, 119991 Moscow, Russia; 3 Molecular Biology and Viral Oncology Unit, Istituto Nazionale Tumori IRCCS Fondazione Pascale, 80131 Naples, Italy; f.buonaguro@istitutotumori.na.it

Infections are responsible for approximately one out of six cases of cancer worldwide [1]. According to the 2018 statistics, each year, as many as 25 cases of cancer per 100,000 people develop due to bacterial or viral infections [2]. Among them, 90% are attributed to the following four agents: *Helicobacter pylori*, human papillomavirus (HPV), and hepatitis B and C viruses (HBV and HCV, respectively). The group of oncogenic viruses also comprises Kaposi’s sarcoma-associated herpesvirus (KSV), the Epstein–Barr virus (EBV), and human T-lymphotropic virus 1 (HTLV-1) [3]. High-risk genotypes of HPVs (HR HPVs) [4] account for 95% of cervical cancer cases [2,5], whereas HBV and HCV account for >75% of all cases of hepatocellular carcinoma worldwide [2]. KSV is the major cause of death for people living with HIV-1 infection (PLWH) with acquired immunodeficiency syndrome (AIDS) or with immunodeficiencies of other origins [6]. Many of these cancer cases could be prevented by either prophylactic vaccination, as in the case of HBV and HPV, or effective therapies, such as the ones in use for *H. pylori* and HCV, which effectively eradicate these infections [7]. Unfortunately, both approaches have insufficient coverage for multiple reasons. For vaccines, it is due to vaccine shortages and vaccine hesitance, and for therapies, to untimely diagnostics, high treatment costs and drug resistance. Altogether, this leads to high death tolls from cancers associated with viral infections [8,9,10]. This Special Issue covers different aspects of this problem.

A series of studies has addressed forms of cancer for which an association with viral infection is still undefined. Dongbin Ahn et al. presented a study on the prevalence and characteristics of HPV infection in oropharyngeal squamous-cell papilloma (OPSP). Although HPV infection has been long since recognized as the primary cause of oropharyngeal cancer, studies on HPV representation in cases of OPSP are lacking. Ahn and co-authors filled this niche in knowledge as they evaluated the prevalence and characteristics of HPV infections in retrospectively enrolled patients with histologically confirmed OPSP. HPV test results were positive in 14.5% of cases (12 of 83 patients), low-risk HPVs were detected in 3.6% of cases, and high-risk HPVs in 10.8% of cases, i.e., in a significantly lower proportion of cases than in the cervical (>99% [11]) or anal cancer (>80%, [12]). The data collected by Ahn et al. support the results of the earlier studies of cancers of the oral cavity, where <10% of cases (mainly cancer of the tongue) were associated with an HR HPV infection [13]. This group has previously shown nearly the same low positivity for HR HPVs in the tonsillar tissues of tumor-free patients (approx. 2%), similar in the adult and pediatric populations [14]. In all these studies, the most prevalent genotype was HPV16.

The HR HPV oncoprotein E7 directly interacts with the retinoblastoma protein (pRb). As a tumor-suppressor protein, pRb binds to E2F transcription factors, which are the key regulators of cell cycle progression, namely transcription factor-transcribing S-phase proteins. Binding to E7 causes the degradation of pRb and the release of E2F factors, resulting in uncontrolled cell division [15]. One of the E2F factors is the tumor-suppressor protein p16, which is a regulator of the normal cell cycle with a function of the CDK inhibitor. By inhibiting CDK 4/6 and preventing Rb phosphorylation, p16 slows the progression of the cell cycle from the G1 to the S phase [16]. The immunohistochemically detected overexpression of p16 serves as a reliable surrogate marker of HPV-associated cancer, independent of the genotype of HR HPV involved in cancer development [17,18,19]. Interestingly and contrary to the observations in cervical or anal cancer, Ahn et al. found OPSPs to be negative for p16 expression. Similar findings were made earlier for oral cavity squamous-cell carcinomas (OCSCCs) [13]. Altogether, these data point to the poor association of carcinomas of the oral cavity with HR HPV infection. However, this association cannot be fully ruled out, as certain factors have been shown to potentiate viral involvement in carcinogenesis.

Several studies have addressed the associations between cancer incidence and cancer forms with infections with viruses other than HPV, HBV, and HCV. Cytomegalovirus is one of the members of the Herpesviridae family, which is not considered oncogenic. Still, persistent infection with HCMV is associated with carcinogenesis [20]. It is often found in tumors of different origins, such as brain tumors and breast, prostate, and colon cancers [21,22,23,24]. Importantly, this virus was reported to be active in the tumor tissues and latent in the normal tissues of the same patients [23,24,25]. HCMV is known to exhibit an immunomodulatory effect, impact the oncogenic pathways, and confer the survival of cancer cells [26,27,28]. Furthermore, some HCMV variants appear to be more oncogenic than others [29]. Overall, data on HCMV oncogenicity are discrepant; the actual role of HCMV in cancer progression remains largely unknown. A contribution by El Baba et al. from the G. Herbein group fills this niche. El Baba et al. demonstrated that HCMV strains isolated from triple-negative breast cancers (TNBCs) can transform human mammary epithelial cells (HMECs) and establish prolonged virus replication. Moreover, the virus conferred infected cells with the capacity for anchorage-independent growth alongside the expression of stem cell markers, i.e., provided cells with the features of aggressive tumor behavior.

The group led by Bandar Alosaimi studied the involvement in cancer of another member of the Herpesviridae family: the Epstein–Barr virus (EBV). EBV belongs to the subfamily Gammaherpesvirinae, the genus Lymphocryptovirus, and the species Human herpesvirus 4. Sequencing-based studies revealed the existence of two EBV types. Over 95% of the adult population between the ages of 35 and 40 are EBV carriers. There is a strong association between EBV infection and Hodgkin’s lymphoma (HL): EBV is present in 40% of HL tumors; however, its role in this disease’s pathogenesis is not fully clarified [30]. In this Special Issue, the study by Al-Anazi A et al. described the role of EBV in the genesis of nasoparyngeal carcinomas (NPCs). Authors found NPC, specifically undifferentiated non-keratinized squamous-cell carcinomas, to be mainly associated with the infection with EBV genotype I, specifically common to Saudi Arabia.

Several studies in this Special Issue address the factors reported to modulate virus involvement in carcinogenesis. Firstly, these are specific “signatures” within the virus. Georges Herbein and co-workers stipulated that transformation-inducing potential, cancer types, and clinical outcomes of the patients depend on the heterogeneity of HCMV strains, with distinct strains responsible for distinct viral properties. They evaluated the molecular features, in vitro transforming potential, and cellular phenotypes acquired by cells infected with a panel of HCMV strains. Certain strains were capable of sustained replication, induced an overexpression of Myc, and established an epithelial/mesenchymal hybrid state of the infected cells. The oncogenic and stemness signatures of HCMV strains accentuate the oncogenic potential of HCMV, suggesting the possibilities of personalized targeted therapies for cancers associated with certain HCMV infections.

Interesting findings were made by Michael Dean and coworkers, who, on the contrary, found viral signatures affecting the expression of viral oncoproteins and hampering virus-induced transformation. In an earlier study by the group, Dean and co-authors observed significantly more E7 amino acid-altering variants in conditionally healthy controls than in cancer cases [30]. To investigate if these observations can be explained by differences in the function(s) of E7 variants, Dean et al. constructed full-length HPV16 E7 genes with amino acid substitutions H9R, D21N, N29S, E33K, T56I, D62N, S63F, S63P, T64M, E80K, D81N, P92L, and P92S, which were found earlier only among healthy controls. Furthermore, E7 variants with D14E, N29H were found in cervical intraepithelial neoplasia grade 2 (CIN2) and P6L, H51N, and R77S were found in CIN3. Firstly, they determined the steady-state level of cytoplasmic and nuclear forms of HPV16 E7. The variants with substitutions found among the controls demonstrated reduced levels of E7 expression, which were in part concomitant with the reduced protein levels of E7. On the contrary, variants with CIN3 amino acid substitutions exhibited levels of E7 similar to those exhibited by thewild-type E7. Furthermore, the “healthy control” H9R, E33K, P92L and P92S variants of E7 had a lower transforming activity in NIH3T3 cells than CIN2 E7 variants with the D14E and N29H or CIN3 variant with R77S. The CIN3 R77S E7 variant also caused an increased migration of NIH3T3 cells in the wound-healing assay compared to the “healthy control” and CIN2 E7 variants. This study demonstrates the importance of fully active E7 in cancer development. Furthermore, it provides experimental proof of the crucial role of viral correlates of oncogenicity and points to the possibility of predicting the clinical course and outcome of HR HPV infection using viral signatures.

There are also multiple other factors enhancing virus involvement in tumorigenesis. For HR HPVs, an important aggravating factor are co-infections, specifically infections with the human immunodeficiency virus (HIV-1) and other viruses, such as Kaposi sarcoma-associated herpesvirus (KSHV) [31]. In people living with HIV-1 (PLWH), the incidence of anal cancer associated with HR HPV infection is steadily increasing despite the success of ART. Interestingly, whereas HPV 16 is the most common genotype in anal high-grade squamous intraepithelial lesions and squamous-cell cancers in the general population, in PLWH it is less common, more prevalent are other non-HPV 16 HR HPV types [32]. Furthermore, infection with multiple HR genotypes is a hallmark for all PLWH tissues, not only cancerous but also benign ones (https://www.mdpi.com/2072-6694/15/3/660). 

There is an intriguing aspect of virus involvement in carcinogenesis other than the potentiation of tumorigenesis, namely the possibility of cancer cells to serve as an immune-privileged niche for viruses which otherwise would have been cleared by the immune system. This possibility was investigated by Smirnova et al. for the infection with the coronovirus SARS-CoV-2. The SARS-CoV-2 pandemic generated extensive knowledge of this infectious agent. SARS-CoV-2 is responsible for the coronavirus disease (COVID-19): a complex of syndromes including very strong inflammation and hypercoagulation disorders. Although this virus primarily infects respiratory and gastrointestinal tracts [33,34], there are many reports demonstrating that it can also replicate in various types of cells of the central nervous system [35], heart [36,37], pancreas [38], kidneys [39] and liver [40]. Smirnova et al. demonstrated that SARS-CoV-2 does not infect any non-transformed liver cell lines but readily infects hepatocarcinoma cell lines and, importantly, some low-passage variants of the glioblastoma multiforme. In fact, the most permissive for the infection were the glioblastoma cell lines. The level of SARS-CoV-2 replication in these cell lines was even higher than in Vero E6 cells, which are the gold standard for virus research due to their high capacity to support the replication of SARS-CoV-2. Single case reports suggest that, in some cases, the coronavirus can suppress tumor growth (for example, [41,42]). The study by Smirnova et al. did not reveal any signs of the cytopathic effect of SARS-CoV-2 in infected hepatocarcinoma or glioblastoma cells. Their data argue against the possibility to use SARS-CoV-2 for the virotherapy of cancer. On the contrary, it indicates that cancer patient’s tumor cells can act as a reservoir supporting SARS-CoV-2’s persistence.

Mechanisms by which viruses modulate malignant transformation of the cell are multiple. It was unequivocally shown that the altered production and scavenging of reactive oxygen species (ROSs) augments inflammation, targeting various redox-sensitive transcription factors and signaling proteins [43]. Viruses are actively involved in these processesby (i) affecting DNA repair and the maintenance of telomere length, (ii) interfering with signaling pathways that control cell cycle and proliferation, and (iii) inducing inflammation. All these processes lead to DNA damage/chromosomal instability [3,44]. This research angle is addressed in the review by Maria Lina Tornesello et al., which focuses on the capacity of human oncoviruses to subvert the function of telomerase reverse transcriptase (TERT). TERT is a catalytic subunit of telomerase and plays a key role in chromosomal stability by maintaining telomere length and allowing cells to avert senescence [45]. TERT activity is universally enhanced in virus-related cancers. A series of earlier studies by the same group identified specific mutations in the TERT promoter, serving to increase TERT expression, not necessarily dependent on the viral infection [46,47]. Viruses have a more complex approach to the modulation of TERT’s expression and activity than just inducing (or favoring) the mutations of TERT promoter. Namely, viral oncoproteins interact with regulatory elements in the infected cells. Specifically, they bind to the TERT promoter and cause the transcriptional activation of the TERT gene. They can also induce post-transcriptional alterations of TERT mRNA as well as epigenetic modifications, which have important effects on the regulation of telomeric and extra-telomeric functions of the telomerase. The list ofviral oncoproteins with these properties includes E6 of HR HPVs, the hepatitis C virus nucleocapsid (core) protein, hepatitis B virus HBVx, the latent membrane protein 1 (LMP1) protein of EBV, Kaposi’s sarcoma-associated herpesvirus (HHV-8) LANA and HTLV-1 transactivator protein Tax. Viruses, such as herpesviruses and HR HPVs, can also operate by integrating their genomes within telomeres or inducing an alternative lengthening of the telomeres. The review by Maria Lina Tornesello and co-authors recapitulates the recent findings in this interplay between oncogenic viruses and telomerase as well as oncogenic pathways activated by oncogenic viruses.

The virus-modulated driving of tumorigenesis is also tightly linked to the perturbance of metabolic pathways in the infected cells [48]. Several viruses, such as HCV and HBV, HR HPVs, and oncogenic herpes viruses, hijack the cell metabolism and remodel it to facilitate carcinogenesis [49,50,51]. Previously, Birke Bartosch’s group gave an overview of how oncogenic viruses perturb metabolic pathways in infected cells [52]. In this Special Issue, Gaballah and Bartosch present a review updating the data accumulated in this field. Such in-depth understanding of the metabolic regulation of cancer cells paves the way for new methods of cancer treatment [53]. This concept is elegantly illustrated in this Special Issue by Chemin et al., who reviewed the role of poly(ADP-ribose) polymerase (PARP) in the repair of double-stranded DNA breaks (DSDBs) in cancer. Previously, Chemin’s group observed high levels of the surrogate DNA damage marker gammaH2AX in hepatocellular carcinomas (HCCs) associated with HBV and HCV infection compared to the peritumoral and control tissues [54]. Elevated levels of gammaH2AX in HCC tumor tissues were also reported in other studies [55,56], altogether indicating the importance of DSDB repair for the survival of cancer cells. The inhibition of the repair of DSDBs could compromise it, specifically under radiotherapy. The inhibition of PARP could also serve to block its other pro-carcinogenic activities, such as the promotion of the transcription of numerous oncogenes, such as c-Myc and Cyclin D1 [57]. In view of this, the use of PARP inhibitors and PARP decoys as radiosensitizing drugs is an option for the treatment of HCCs specifically associated with HBV infection. Overall, both the replication of oncogenic viruses and the proliferation of infected cells acquire dependency on a wide variety of intracellular pathways, presenting targets for therapeutic interventions.

Another intriguing option of treatment of virus-associated cancers is therapeutic vaccination against viral antigens expressed by the cancer cells. The most progress that has been made in this field has been achieved in therapeutic vaccination against HR HPV-associated neoplasia and cancer, specifically cervical cancer. Several trials, such as the trial described by Trimble CL et al. [58], demonstrating significant therapeutic effects manifested by the reversal of lesions and/or decrease in the virus load or viral clearance. By now, researchers have concluded and published an approach to review the results of the therapeutic vaccination studies as a treatment of high-grade cervical lesions associated with HPV16 infection, along with a co plex assessment of their safety, efficacy, and immunogenicity [59]. In this Special Issue, Gonçalves CA and co-authors presented the results of such systematic review. Overall, the therapeutic vaccines against HR HPVs were heterogeneous in their formulation, dose, intervention protocol, and routes of administration. Of the total of 1184 studies identified, only 16 made a complex assessment of: (i) the adverse/toxic effects associated with the therapeutic vaccine administration; (ii) histopathological regression of the lesion and/or regression of the lesion size and/or viral clearance; (iii) the immunological response of individuals who received treatment compared to those who did not or before and after receiving the vaccine. Most of these studies (15/16) showed HR HPV vaccines to be safe and well tolerated, with clinical efficacy regarding the lesions and histopathological regression or viral clearance. The summarized results of these trials point at therapeutic vaccination as a promising treatment endpoint for HR HPV induced cervical lesions.

Concluding the topic of vaccines against virus-associated cancer is the review by Margaret Liu on the rationale and mechanisms of action of these vaccines and the progress made in their development. This review dwells on the earlier analysis by Liu et al. demonstrating the crucial role of nucleic acid (NA) vaccines for One Health, showing the key aspects of the development of plasmid DNA vaccines (which were earlier licensed for a number of veterinary uses and tested against human viral infections, such as HIV-1, HCV, HPV) to illustrate how they helped pave the way for the mRNA vaccines of today [60,61]. Interest in NA vaccines, based on DNA and mRNA, for both prophylactic and therapeutic uses, has greatly increased following the successful deployment of two mRNAs and, on a more limited scale, one DNA vaccine for COVID-19. Great efforts are being made toward using them to treat chronic infections and cancer. The review examines the past and current successes of such therapies using other technologies and assesses the characteristics of DNA and mRNA vaccines, which could eventually turn them into attractive therapies for chronic viral infections and cancer and an alternative to more traditional chemo- and immunotherapies.

## References

[1] Plummer M., de Martel C., Vignat J., Ferlay J., Bray F., Franceschi S. (2016). Global burden of cancers attributable to infections in 2012: A synthetic analysis. Lancet Glob. Health.

[2] de Martel C., Georges D., Bray F., Ferlay J., Clifford G.M. (2020). Global burden of cancer attributable to infections in 2018: A worldwide incidence analysis. Lancet Glob. Health.

[3] Krump N.A., You J. (2018). Molecular mechanisms of viral oncogenesis in humans. Nat. Rev. Microbiol..

[4] National Cancer Institute. https://www.cancer.gov/about-cancer/causes-prevention/risk/infectious-agents/hpv-and-cancer.

[5] WHO. https://www.who.int/news-room/fact-sheets/detail/human-papilloma-virus-and-cancer.

[6] Minhas V., Wood C. (2014). Epidemiology and transmission of Kaposi’s sarcoma-associated herpesvirus. Viruses.

[7] Rositch A.F. (2020). Global burden of cancer attributable to infections: The critical role of implementation science. Lancet Glob. Health.

[8] Debarry J., Cornberg M., Manns M.P. (2017). Challenges in warranting access to prophylaxis and therapy for hepatitis B virus infection. Liver Int..

[9] Sung H., Ferlay J., Siegel R.L., Laversanne M., Soerjomataram I., Jemal A., Bray F. (2021). Global Cancer Statistics 2020: GLOBOCAN Estimates of Incidence and Mortality Worldwide for 36 Cancers in 185 Countries. CA Cancer J. Clin..

[10] Ebrahimi N., Yousefi Z., Khosravi G., Malayeri F.E., Golabi M., Askarzadeh M., Shams M.H., Ghezelbash B., Eskandari N. (2023). Human papillomavirus vaccination in low- and middle-income countries: Progression, barriers, and future prospective. Front. Immunol..

[11] Okunade K.S. (2020). Human papillomavirus and cervical cancer. J. Obs. Gynaecol..

[12] Lin C., Franceschi S., Clifford G.M. (2018). Human papillomavirus types from infection to cancer in the anus, according to sex and HIV status: A systematic review and meta-analysis. Lancet Infect. Dis..

[13] Katirachi S.K., Gronlund M.P., Jakobsen K.K., Gronhoj C., von Buchwald C. (2023). The Prevalence of HPV in Oral Cavity Squamous Cell Carcinoma. Viruses.

[14] Ahn D., Heo S.J., Lee G.J., Sohn J.H., Jeong J.Y. (2022). Prevalence and characteristics of tonsillar human papillomavirus infection in tumor-free patients undergoing tonsillectomy. Auris Nasus Larynx.

[15] Pal A., Kundu R. (2019). Human Papillomavirus E6 and E7: The Cervical Cancer Hallmarks and Targets for Therapy. Front. Microbiol..

[16] Cosper P.F., Bradley S., Luo L., Kimple R.J. (2021). Biology of HPV Mediated Carcinogenesis and Tumor Progression. Semin. Radiat. Oncol..

[17] Nicolas I., Saco A., Barnadas E., Marimon L., Rakislova N., Fuste P., Rovirosa A., Gaba L., Bunesch L., Gil-Ibanez B. (2020). Prognostic implications of genotyping and p16 immunostaining in HPV-positive tumors of the uterine cervix. Mod. Pathol..

[18] Amin F.A.S., Un Naher Z., Ali P.S.S. (2023). Molecular markers predicting the progression and prognosis of human papillomavirus-induced cervical lesions to cervical cancer. J. Cancer Res. Clin. Oncol..

[19] Dovnik A., Repse Fokter A. (2023). The Role of p16/Ki67 Dual Staining in Cervical Cancer Screening. Curr. Issues Mol. Biol..

[20] Yu C., He S., Zhu W., Ru P., Ge X., Govindasamy K. (2023). Human cytomegalovirus in cancer: The mechanism of HCMV-induced carcinogenesis and its therapeutic potential. Front. Cell. Infect. Microbiol..

[21] Wolmer-Solberg N., Baryawno N., Rahbar A., Fuchs D., Odeberg J., Taher C., Wilhelmi V., Milosevic J., Mohammad A.A., Martinsson T. (2013). Frequent detection of human cytomegalovirus in neuroblastoma: A novel therapeutic target?. Int. J. Cancer.

[22] Harkins L., Volk A.L., Samanta M., Mikolaenko I., Britt W.J., Bland K.I., Cobbs C.S. (2002). Specific localisation of human cytomegalovirus nucleic acids and proteins in human colorectal cancer. Lancet.

[23] Samanta M., Harkins L., Klemm K., Britt W.J., Cobbs C.S. (2003). High prevalence of human cytomegalovirus in prostatic intraepithelial neoplasia and prostatic carcinoma. J. Urol..

[24] Harkins L.E., Matlaf L.A., Soroceanu L., Klemm K., Britt W.J., Wang W., Bland K.I., Cobbs C.S. (2010). Detection of human cytomegalovirus in normal and neoplastic breast epithelium. Herpesviridae.

[25] Cobbs C.S., Harkins L., Samanta M., Gillespie G.Y., Bharara S., King P.H., Nabors L.B., Cobbs C.G., Britt W.J. (2002). Human cytomegalovirus infection and expression in human malignant glioma. Cancer Res..

[26] Vinogradskaya G.R., Ivanov A.V., Kushch A.A. (2022). Mechanisms of Survival of Cytomegalovirus-Infected Tumor Cells. Mol. Biol..

[27] Kumar A., Tripathy M.K., Pasquereau S., Al Moussawi F., Abbas W., Coquard L., Khan K.A., Russo L., Algros M.P., Valmary-Degano S. (2018). The Human Cytomegalovirus Strain DB Activates Oncogenic Pathways in Mammary Epithelial Cells. EBioMedicine.

[28] Michaelis M., Doerr H.W., Cinatl J. (2009). The story of human cytomegalovirus and cancer: Increasing evidence and open questions. Neoplasia.

[29] Herbein G. (2022). High-Risk Oncogenic Human Cytomegalovirus. Viruses.

[30] Gequelin L.C., Riediger I.N., Nakatani S.M., Biondo A.W., Bonfim C.M. (2011). Epstein-Barr virus: General factors, virus-related diseases and measurement of viral load after transplant. Rev. Bras. Hematol. Hemoter..

[31] Dai L., Zhao M., Jiang W., Lin Z., Del Valle L., Qin Z. (2018). KSHV co-infection, a new co-factor for HPV-related cervical carcinogenesis?. Am. J. Cancer Res..

[32] Chowdhury S., Darragh T.M., Berry-Lawhorn J.M., Isaguliants M.G., Vonsky M.S., Hilton J.F., Lazar A.A., Palefsky J.M. (2023). HPV Type Distribution in Benign, High-Grade Squamous Intraepithelial Lesions and Squamous Cell Cancers of the Anus by HIV Status. Cancers.

[33] Lamers M.M., Beumer J., van der Vaart J., Knoops K., Puschhof J., Breugem T.I., Ravelli R.B.G., Paul van Schayck J., Mykytyn A.Z., Duimel H.Q. (2020). SARS-CoV-2 productively infects human gut enterocytes. Science.

[34] Zhu N., Zhang D., Wang W., Li X., Yang B., Song J., Zhao X., Huang B., Shi W., Lu R. (2020). A Novel Coronavirus from Patients with Pneumonia in China, 2019. N. Engl. J. Med..

[35] Crunfli F., Carregari V.C., Veras F.P., Silva L.S., Nogueira M.H., Antunes A., Vendramini P.H., Valenca A.G.F., Brandao-Teles C., Zuccoli G.D.S. (2022). Morphological, cellular, and molecular basis of brain infection in COVID-19 patients. Proc. Natl. Acad. Sci. USA.

[36] Sharma A., Garcia G., Wang Y., Plummer J.T., Morizono K., Arumugaswami V., Svendsen C.N. (2020). Human iPSC-Derived Cardiomyocytes Are Susceptible to SARS-CoV-2 Infection. Cell Rep. Med..

[37] Bailey A.L., Dmytrenko O., Greenberg L., Bredemeyer A.L., Ma P., Liu J., Penna V., Winkler E.S., Sviben S., Brooks E. (2021). SARS-CoV-2 Infects Human Engineered Heart Tissues and Models COVID-19 Myocarditis. JACC Basic Transl. Sci..

[38] Muller J.A., Gross R., Conzelmann C., Kruger J., Merle U., Steinhart J., Weil T., Koepke L., Bozzo C.P., Read C. (2021). SARS-CoV-2 infects and replicates in cells of the human endocrine and exocrine pancreas. Nat. Metab..

[39] Diao B., Wang C., Wang R., Feng Z., Zhang J., Yang H., Tan Y., Wang H., Wang C., Liu L. (2021). Human kidney is a target for novel severe acute respiratory syndrome coronavirus 2 infection. Nat. Commun..

[40] Wanner N., Andrieux G., Badia I.M.P., Edler C., Pfefferle S., Lindenmeyer M.T., Schmidt-Lauber C., Czogalla J., Wong M.N., Okabayashi Y. (2022). Molecular consequences of SARS-CoV-2 liver tropism. Nat. Metab..

[41] Pasin F., Mascalchi Calveri M., Calabrese A., Pizzarelli G., Bongiovanni I., Andreoli M., Cattaneo C., Rignanese G. (2020). Oncolytic effect of SARS-CoV2 in a patient with NK lymphoma. Acta Biomed..

[42] Bounassar-Filho J.P., Boeckler-Troncoso L., Cajigas-Gonzalez J., Zavala-Cerna M.G. (2023). SARS-CoV-2 as an Oncolytic Virus Following Reactivation of the Immune System: A Review. Int. J. Mol. Sci..

[43] Lennicke C., Cocheme H.M. (2021). Redox metabolism: ROS as specific molecular regulators of cell signaling and function. Mol Cell.

[44] Tornesello M.L., Annunziata C., Tornesello A.L., Buonaguro L., Buonaguro F.M. (2018). Human Oncoviruses and p53 Tumor Suppressor Pathway Deregulation at the Origin of Human Cancers. Cancers.

[45] Daniel M., Peek G.W., Tollefsbol T.O. (2012). Regulation of the human catalytic subunit of telomerase (hTERT). Gene.

[46] Annunziata C., Pezzuto F., Greggi S., Ionna F., Losito S., Botti G., Buonaguro L., Buonaguro F.M., Tornesello M.L. (2018). Distinct profiles of TERT promoter mutations and telomerase expression in head and neck cancer and cervical carcinoma. Int. J. Cancer.

[47] Pezzuto F., Izzo F., De Luca P., Biffali E., Buonaguro L., Tatangelo F., Buonaguro F.M., Tornesello M.L. (2021). Clinical Significance of Telomerase Reverse-Transcriptase Promoter Mutations in Hepatocellular Carcinoma. Cancers.

[48] Ivanov A.V., Valuev-Elliston V.T., Tyurina D.A., Ivanova O.N., Kochetkov S.N., Bartosch B., Isaguliants M.G. (2017). Oxidative stress, a trigger of hepatitis C and B virus-induced liver carcinogenesis. Oncotarget.

[49] Levy P.L., Duponchel S., Eischeid H., Molle J., Michelet M., Diserens G., Vermathen M., Vermathen P., Dufour J.F., Dienes H.P. (2017). Hepatitis C virus infection triggers a tumor-like glutamine metabolism. Hepatology.

[50] Thaker S.K., Ch’ng J., Christofk H.R. (2019). Viral hijacking of cellular metabolism. BMC Biol..

[51] Mullen P.J., Christofk H.R. (2022). The Metabolic Relationship between Viral Infection and Cancer. Annu. Rev. Cancer Biol..

[52] Levy P., Bartosch B. (2016). Metabolic reprogramming: A hallmark of viral oncogenesis. Oncogene.

[53] Oliveira G.L., Coelho A.R., Marques R., Oliveira P.J. (2021). Cancer cell metabolism: Rewiring the mitochondrial hub. Biochim. Biophys. Acta Mol. Basis Dis..

[54] Gerossier L., Dubois A., Paturel A., Fares N., Cohen D., Merle P., Lachuer J., Wierinckx A., Saintigny P., Bancel B. (2021). PARP inhibitors and radiation potentiate liver cell death in vitro. Do hepatocellular carcinomas have an achilles’ heel?. Clin. Res. Hepatol. Gastroenterol..

[55] Evert M., Frau M., Tomasi M.L., Latte G., Simile M.M., Seddaiu M.A., Zimmermann A., Ladu S., Staniscia T., Brozzetti S. (2013). Deregulation of DNA-dependent protein kinase catalytic subunit contributes to human hepatocarcinogenesis development and has a putative prognostic value. Br. J. Cancer.

[56] Xiao H., Tong R., Ding C., Lv Z., Du C., Peng C., Cheng S., Xie H., Zhou L., Wu J. (2015). gamma-H2AX promotes hepatocellular carcinoma angiogenesis via EGFR/HIF-1alpha/VEGF pathways under hypoxic condition. Oncotarget.

[57] Idogawa M., Yamada T., Honda K., Sato S., Imai K., Hirohashi S. (2005). Poly(ADP-ribose) polymerase-1 is a component of the oncogenic T-cell factor-4/beta-catenin complex. Gastroenterology.

[58] Trimble C.L., Morrow M.P., Kraynyak K.A., Shen X., Dallas M., Yan J., Edwards L., Parker R.L., Denny L., Giffear M. (2015). Safety, efficacy, and immunogenicity of VGX-3100, a therapeutic synthetic DNA vaccine targeting human papillomavirus 16 and 18 E6 and E7 proteins for cervical intraepithelial neoplasia 2/3: A randomised, double-blind, placebo-controlled phase 2b trial. Lancet.

[59] Goncalves C.A., Lopes-Junior L.C., Nampo F.K., Zilly A., Mayer P.C.M., Pereira-da-Silva G. (2019). Safety, efficacy and immunogenicity of therapeutic vaccines in the treatment of patients with high-grade cervical intraepithelial neoplasia associated with human papillomavirus: A systematic review protocol. BMJ Open.

[60] Fomsgaard A., Liu M.A. (2021). The Key Role of Nucleic Acid Vaccines for One Health. Viruses.

[61] Knezevic I., Liu M.A., Peden K., Zhou T., Kang H.N. (2021). Development of mRNA Vaccines: Scientific and Regulatory Issues. Vaccines.

